# Real-Time Evolution of Zika Virus Disease Outbreak, Roatán, Honduras

**DOI:** 10.3201/eid2308.161944

**Published:** 2017-08

**Authors:** Trevor Brooks, Arup Roy-Burman, Cascade Tuholske, Michael P. Busch, Sonia Bakkour, Mars Stone, Jeffrey M. Linnen, Kui Gao, Jayleen Coleman, Evan M. Bloch

**Affiliations:** University of California School of Medicine, San Francisco, California, USA (T. Brooks, A. Roy-Burman, M.P. Busch);; University of California, Santa Barbara, California, USA (C. Tuholske);; Blood Systems Research Institute, San Francisco (M.P. Busch, S. Bakkour, M. Stone, E.M. Bloch);; Hologic Inc., San Diego, California, USA (J.M. Linnen, K. Gao);; Public Hospital Roatán, Coxen Hole, Honduras (J. Coleman);; Johns Hopkins School of Medicine, Baltimore, Maryland, USA (E.M. Bloch)

**Keywords:** Zika virus, Zika virus disease, viruses, outbreak, infection, evolution, dengue, dengue virus, chikungunya, chikungunya virus, epidemiology, arboviruses, flaviviruses, vector-borne infections, Roatán, Honduras

## Abstract

A Zika virus disease outbreak occurred in Roatán, Honduras, during September 2015–July 2016. Blood samples and clinical information were obtained from 183 patients given a clinical diagnosis of suspected dengue virus infection. A total of 79 patients were positive for Zika virus, 13 for chikungunya virus, and 6 for dengue virus.

Zika virus is a mosquitoborne flavivirus that is clinically nonspecific and associated with severe congenital injury (*1*–*5*). Zika virus is closely related to dengue virus (DENV), which is endemic to Roatán, the largest Honduran Bay island (population ≈46,000 persons), located 50 km north of the Honduras mainland (*6*). However, most cases of DENV infection are diagnosed clinically because of limited capacity for laboratory confirmation.

We initiated a pilot study in 2015 to correlate clinical (signs and symptoms) and laboratory diagnostic findings for cases of presumptive dengue. As of September 2015, Zika virus had not been identified in Roatán. We report emergence of Zika virus on Roatán.

## The Study

We conducted a cross-sectional survey of patients who came to Public Hospital Roatán during September 2015–July 2016 and were given a clinical diagnosis of suspected dengue fever. This hospital, the only public hospital in Roatán, has 58 inpatient beds. Approximately 90% of dengue cases on Roatán are diagnosed at this hospital; most case-patients came to the emergency department, which had 18,578 visits in 2015 (Public Hospital Roatán Internal Statistics, unpub. data).

We used a convenience sampling approach. All patients >2 years of age given a diagnosis of clinically suspected DENV infection at the hospital during the enrollment period were eligible. Suspected DENV infection was diagnosed by using criteria of the Honduran Ministry of Health, which are fever plus 2 of the following: nausea/vomiting, rash, headache/retroorbital pain, myalgias/arthralgias, petechiae/positive tourniquet test result, leukopenia, and bleeding.

Patients were enrolled after we obtained informed consent; minors were enrolled only after we obtained parental permission. Ethical approval was granted by institutional review boards of the University of California, San Francisco, and Universidad Nacional Autónoma de Honduras. Enrollment of participants and blood collection were restricted to workday hours (8:00 am–4:00 pm) Monday through Friday. Patients underwent a phlebotomy and completed a clinical questionnaire, administered verbally in English or Spanish that addressed demographics, migration history, employment, medical history, and symptoms and signs of arbovirus infection.

Samples of whole blood (5–9 mL) were collected in EDTA-treated vacutainers (BD Diagnostics, Franklin Lakes, NJ, USA) within 1 h of diagnosis. Samples were centrifuged (35,000 rpm), which yielded sufficient plasma to prepare 5 equal aliquots (minimum 0.5 mL/aliquot). Aliquots were labeled with patient identification numbers and stored frozen at −30°C pending for testing.

In July 2016, samples were shipped on dry ice to Blood Systems Research Institute (San Francisco, CA, USA) where initial testing was performed by using the Trioplex Assay (Centers for Disease Control and Prevention, Atlanta, GA, USA) for detection of DENV, chikungunya virus (CHIKV), and Zika virus RNA. RNA was extracted from 140 μL of plasma and eluted in 60 μL of buffer (QIAamp Viral RNA Mini Kit; QIAGEN, Valencia, CA, USA).

Multiplex real-time reverse transcription PCR (RT-PCR) was performed using the SuperScript III Platinum One-Step qRT-PCR Kit (ThermoFisher Scientific, Pittsburgh, PA, USA) with DENV, CHIKV, and Zika virus primers and probes developed at the Centers for Disease Control and Prevention. Each duplicate reaction contained 10 μL of sample RNA in a reaction volume of 25 µL. Samples were tested in a 96-well format in a real-time instrument (LightCycler 480 System; Roche, Basel. Switzerland). Results were considered positive if the cycle threshold was <38.

A more sensitive Zika virus–only test based on transcription-mediated amplification (Aptima Zika Virus Assay; Hologic Inc., San Diego, CA, USA), which processed 0.5-mL plasma into each amplification reaction, was performed in parallel for all samples to confirm Zika virus infections detected by the Trioplex Assay and detect low levels of Zika virus RNA (*7*). The Aptima Assay was used for further analysis.

At conclusion of study enrollment, addresses of participants were mapped by using a hand-held eTrex 20 (Garmin, Lenexa, KS, USA), which generated global positioning system coordinates for their homes. The survey, laboratory test data, and global positioning system coordinates were uploaded into ArcGIS version 10.3.1 software (Esri, Inc., Redlands, CA, USA). All potential predictors of Zika virus infection were analyzed by using univariate logistic regression models in Stata version 13 (StataCorp LLC, College Station, TX, USA). Characteristics that were significant (p = 0.10) by univariate analysis were entered into a multivariable logistic regression model.

A total of 183 patients participated and provided blood samples (Table 1). Most (60%) patients were women. Mean age was 26 (interquartile range 19–37 years). Mean time to seeking treatment after onset of signs or symptoms was 3.2 days. The most commonly reported signs or symptoms were headache (90%), arthralgia (89%), myalgia (87%), retroorbital pain (71%), and rash (55%). Most patients lived in homes that had a nondirt floor (93%), running water (81%), and electricity (90%). Only 25% reported having mosquito nets over their beds. Six (3%) of 183 patients were positive for DENV RNA, and 13 (75%) were positive for CHIKV RNA. In contrast, Zika virus RNA was detectable in 66 (36%) of 183 patients by the Trioplex assay and in 79 (43%) of 183 patients by the Aptima assay.

**Table 1:** Demographic characteristics of persons with suspected Zika virus infections, Roatán, Honduras, September 2015–July 2016*

Characteristic	Total, n = 183	Zika virus infection, n = 79	No Zika virus infection, n = 104
Age, y	26 (19–37)	27 (21–38)	24 (17–35)
Sex			
M	73 (39.9)	28 (35.4)	45 (43.3)
F	110 (60.1)	51 (64.6)	59 (56.7)
Living conditions			
Dirt floor	12 (6.6)	4 (5.1)	8 (7.7)
Running water	149 (81.4)	71 (89.9)	78 (75.0)
Electricity	165 (90.2)	75 (94.9)	90 (86.5)
Mosquito nets over beds	46 (25.1)	22 (27.8)	24 (23.1)
Rooms	2 (1–2)	2 (1–3)	2 (1–2)
Persons in household	4 (3–5.5)	4 (3–5)	4 (3–6)
Sign or symptoms			
Headache	165 (90.2)	75 (94.9)	90 (86.5)
Muscle aches	159 (86.9)	67 (84.8)	92 (88.5)
Joint ache	162 (88.5)	71 (89.9)	91 (87.5)
Eye pain	131 (71.6)	61 (77.2)	70 (67.3)
Rash	102 (55.7)	73 (92.4)	29 (27.9)
Bleeding	1 (0.5)	0	1 (1.0)
Vomiting	49 (26.8)	11 (13.9)	38 (36.5)
Petechiae	5 (2.7)	2 (2.5)	3 (2.9)
Epistaxis	1 (0.5)	0	1 (1.0)
Gingivitis	3 (1.6)	1 (1.3)	2 (1.9)
Other	123 (67.2)	53 (67.1)	70 (67.3)
Body temperature, °C, mean (SD)	37.8 (1.1)	37.3 (0.9)	38.1 (1.1)
Days between symptom onset and seeking treatment	3 (1–4)	3 (1–4.5)	2 (2–4)
History of infectious diseases			
Dengue	45 (24.6)	21 (26.6)	24 (23.1)
Malaria	33 (18.0)	18 (22.8)	15 (14.4)
Chikungunya	61 (33.3)	28 (35.4)	33 (31.7)

*Values are median (IQR) or no. (%) unless indicated otherwise. IQR, interquartile range.

**Figure 1:**
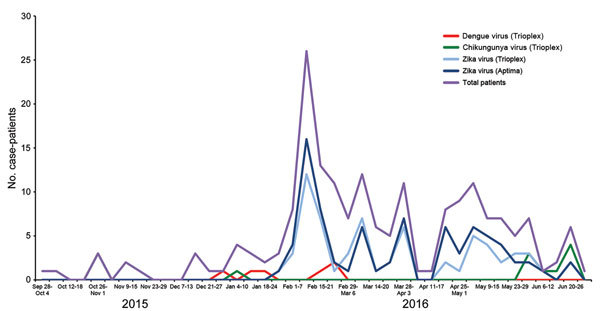
Weekly case occurrence of Zika virus, dengue virus, and chikungunya virus infections, by testing type, Roatán, Honduras, September 2015–July 2016. Aptima, Aptima Zika Virus Assay (Hologic Inc., San Diego, CA, USA); Trioplex, Trioplex Assay (Centers for Disease Control and Prevention, Atlanta, GA, USA).

The enrollment rate was low through the first part of the study, when <3 case-patients/wk were enrolled (Figure 1). In the first week of February 2016, eight case-patients were enrolled, followed by 26 case-patients the following week. The first case of laboratory-confirmed Zika virus infection on Roatán occurred on January 27, 2016; Zika virus infection peaked (16 cases) during February 8–14. Timing of accrual of positive case-patients matched expected seasonality of arbovirus infection. Cases decreased steadily through the remainder of the sample collection period.

**Figure 2:**
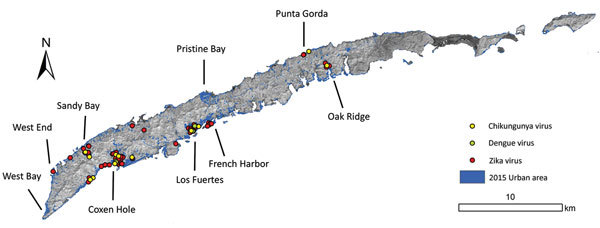
Spatial distribution of confirmed cases of infection with Zika virus, dengue virus, and chikungunya virus, Roatán, Honduras, September 2015–July 2016.

The Zika virus outbreak was focused in the major population centers in Coxen Hole, Los Fuertes, and French Harbor (Figure 2). Except for West Bay and West End, cases were widely distributed on the island. West Bay and West End are major tourist centers on Roatán; absence of cases might be ascribed to tourists and expatriates who were unlikely to seek medical attention at Public Hospital Roatán.

Multivariable analysis showed that rash (odds ratio [OR] 30.6, 95% CI 10.8–86.9) and headache (OR 11.2, 95% CI 2.7–46.7) were independently associated with Zika virus infection. Fever (OR 0.44, 95% CI 0.26–0.74) and vomiting (OR 0.25, 95% CI 0.08–0.73) were associated with a decreased risk for Zika virus infection (Table 2).

**Table 2:** Logistic regression analysis of predictors for Zika virus infection, Roatán, Honduras, September 2015–July 2016

Characteristic	Univariate logistic regression			Multivariable logistic regression	
	Odds ratio (95% CI)	p value		Odds ratio (95% CI)	p value
Household					
Running water	2.96 (1.26–6.96)	0.01		2.93 (0.78–11.1)	0.11
Electricity	2.92 (0.92–9.24)	0.07		2.36 (0.39–14.3)	0.35
Sign or symptom					
Headache	2.92 (0.92–9.24)	0.07		11.20 (2.70–46.7)	0.001
Rash	31.5 (12.3–80.2)	<0.0001		30.6 (10.8–86.9)	<0.0001
Vomiting	0.28 (0.13–0.60)	0.001		0.25 (0.08–0.73)	0.01
Fever	0.38 (0.25–0.57)	<0.0001		0.44 (0.26–0.74)	0.002

This study had limitations. Our exclusive enrollment of patients who came to Public Hospital Roatán excluded patients who came to other healthcare facilities or outside daytime working hours who were not sufficiently symptomatic to seek care. Alternative body fluids (e.g., saliva, urine, whole blood) were not available for testing, and serologic testing for Zika virus was not performed because of cost constraints and cross-reactivity between Zika virus and DENV antibodies. We did not perform confirmatory testing of CHIKV- and DENV-positive samples. This study could be biased for detecting Zika virus and not DENV/CHIKV because of use of a more sensitive assay.

## Conclusions

We demonstrated the evolution of the Zika virus disease outbreak in Roatán, Honduras, in early 2016. Findings highlight challenges in case ascertainment based only on clinical diagnosis. In the absence of laboratory confirmation, clinical diagnosis has low specificity because of overlap of signs and symptoms between infections with Zika virus, DENV, CHIKV, and a host of other regionally endemic infections. Enrollment was contingent upon a suspected diagnosis of dengue. However, DENV was detected in <5% of case-patients, whereas 43% of case-patients had acute Zika virus infections.

After a rapid peak in early February 2016, there was a slow decrease in reported cases through July 2016, when enrollment concluded. Access to laboratory testing remains a barrier to surveillance and clinical management in low-resource countries where Zika virus disease outbreaks are predominantly focused, but laboratory infrastructure is lacking (*2*).
